# Proteomic profiling of human cancer pseudopodia for the identification of anti-metastatic drug candidates

**DOI:** 10.1038/s41598-018-24256-8

**Published:** 2018-04-11

**Authors:** Sunkyu Choi, Aditya M. Bhagwat, Rasha Al Mismar, Neha Goswami, Hisham Ben Hamidane, Lu Sun, Johannes Graumann

**Affiliations:** 1 Research Division, Weill Cornell Medicine - Qatar, Doha, State of Qatar; 2 Present Address: Scientific Service Group Biomolecular Mass Spectrometry, Max Planck Institute for Heart and Lung Research, Bad Nauheim, Germany

## Abstract

Cancer metastasis causes approximately 90% of all cancer-related death and independent of the advancement of cancer therapy, a majority of late stage patients suffers from metastatic cancer. Metastasis implies cancer cell migration and invasion throughout the body. Migration requires the formation of pseudopodia in the direction of movement, but a detailed understanding of this process and accordingly strategies of prevention remain elusive. Here, we use quantitative proteomic profiling of human cancer pseudopodia to examine this mechanisms essential to metastasis formation, and identify potential candidates for pharmacological interference with the process. We demonstrate that Prohibitins (PHBs) are significantly enriched in the pseudopodia fraction derived from cancer cells, and knockdown of PHBs, as well as their chemical inhibition through Rocaglamide (Roc-A), efficiently reduces cancer cell migration.

## Introduction

Cancer metastasis is responsible for over 90% of cancer-related death. In spite of numerous advances in cancer treatment, including surgical techniques, radiation therapy, as well as chemotherapy, a significant number of patients will display metastatic disease regardless of local control. Virtually all cancers are able to metastasize to tissues distant from the tumor of origin, including brain, bone, lungs and liver. The metastatic process is, however, inefficient and relies on several critical steps, of which the entry of cancer cells into the circulatory system constitutes the first step. Thus, poor prognosis is linked to lymph node involvement as well as vascular tumor emboli.

Prominent among genetic changes correlated with invasive characteristics of cancer cells is the capacity to develop specialized protrusive and adhesive cellular structures: pseudopodia, invadopodia and podosomes^1–7^. Invadopodia and podosomes are dynamic actin-based cytoskeleton projections into the plasma membrane and observed on the surface of cells plated on extracellular matrix. They facilitate proteolytic degradation of extracellular matrix by secretion of matrix metalloproteinase^4,5^. In contrast, pseudopodia are larger dynamic actin cytoskeleton-based structures formed at the cell front and promote directional migration in response to chemo-attractive stimuli^3^. Invasive cancer cells produce pseudopodia to penetrate constraining tissue and structures, and migrate e.g. into the lymphatic system and on to distant organs. On the molecular level, several studies report that the Arp2/3 complex, Wave3, Eps8, cortactin, α-actinin, Lim-kinase, fascin, and filamin are associated with pseudopodia formation^8–11^.

Based on these findings from the literature, we hypothesize that pseudopodia-specific proteins associated with regulation of pseudopodia function remain to be discovered and continued characterization of cell protrusions may lead to identification of further players in the development of metastatic disease and thus new therapeutic strategies. Accordingly, we investigated in this study metastatic cancer cell protrusions using deep quantitative proteomic profiling of cancer cell pseudopodia isolated by physical fractionation, in order to identify new target proteins that may be used for blocking cancer cell migration.

## Results

### Extension of Pseudopodia in Response to Serum Stimulation

**Figure 1:**
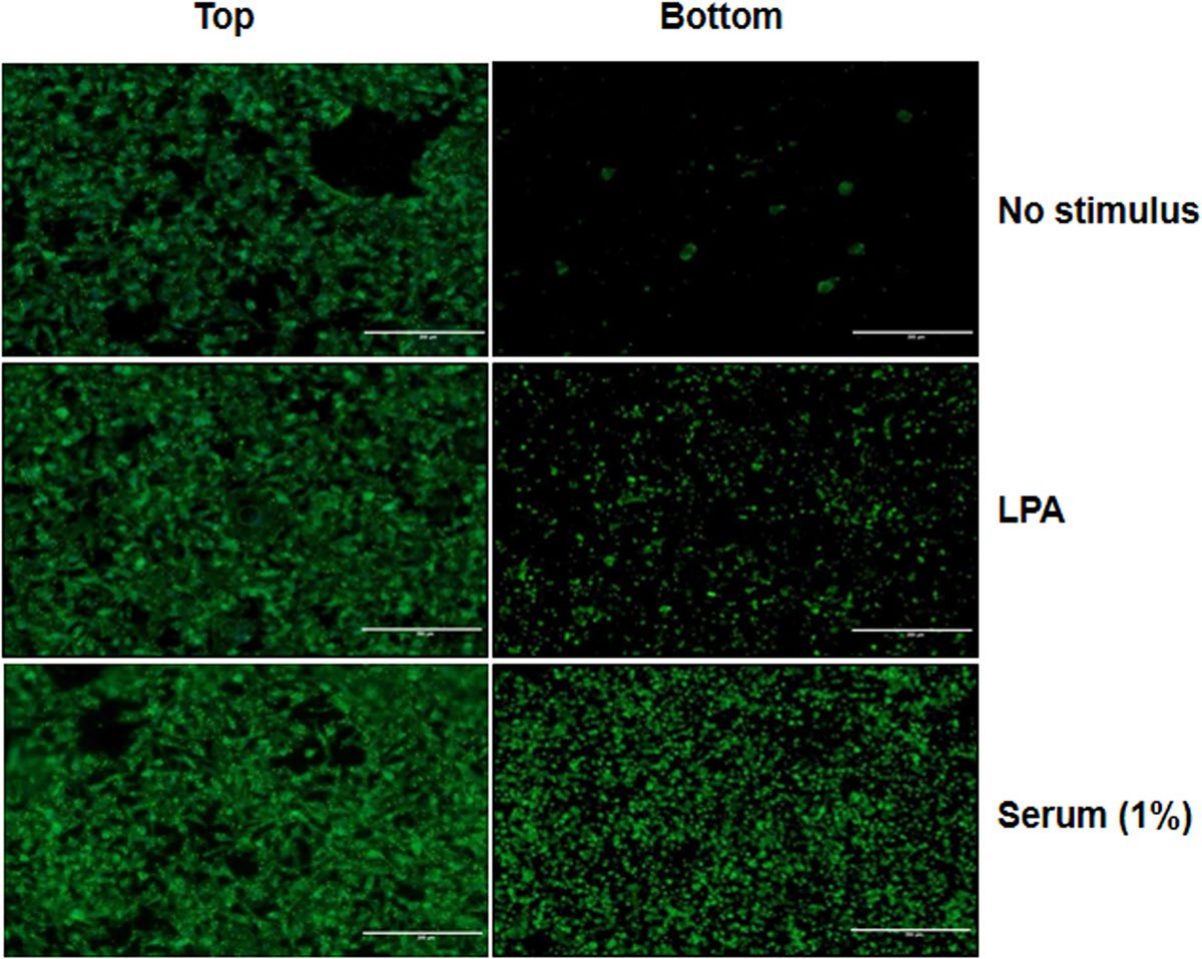
Extension of pseudopodia by MDA-MB-231 cells in response to LPA and serum. Microscope images of MDA-MB-231 cell bodies (top) or pseudopodia (bottom) separated by filters with 3 μm pore size in response to the indicated stimuli. Cells were stained for F-actin with phalloidin. Scale bar = 200 μm.

To establish a model system for the study of pseudopodia in the context of cancer, MDA-MB-231 cells were starved for 16 h, followed by incubation in pseudopodia-stimulating conditions such as LPA or serum stimulation (both in DMEM) and DMEM alone as a control^12–14^. The resulting amount of pseudopodia extension by LPA or serum stimulation was quantified using polycarbonate transwell membrane filters^14^. To prevent cell bodies from penetrating the filter pores, 3-μm pore size was employed to prevent nuclear passage. To image pseudopodia and cell body, the top and bottom of the membrane filters were stained using phalloidin. Figure 1 shows that MDA-MB-231 cells extended pseudopodia through the filters in response to LPA or serum stimulation, but not when DMEM was used alone. Cultures exposed to serum resulted in superior numbers of pseudopodia as compared to LPA. Further experiments and deep quantitative proteomic profiling of pseudopodia were thus performed using 1% serum stimulation.

### Comparative Quantitative Proteomic Analysis of Pseudopodia and Cell Body Fractions

**Figure 2:**
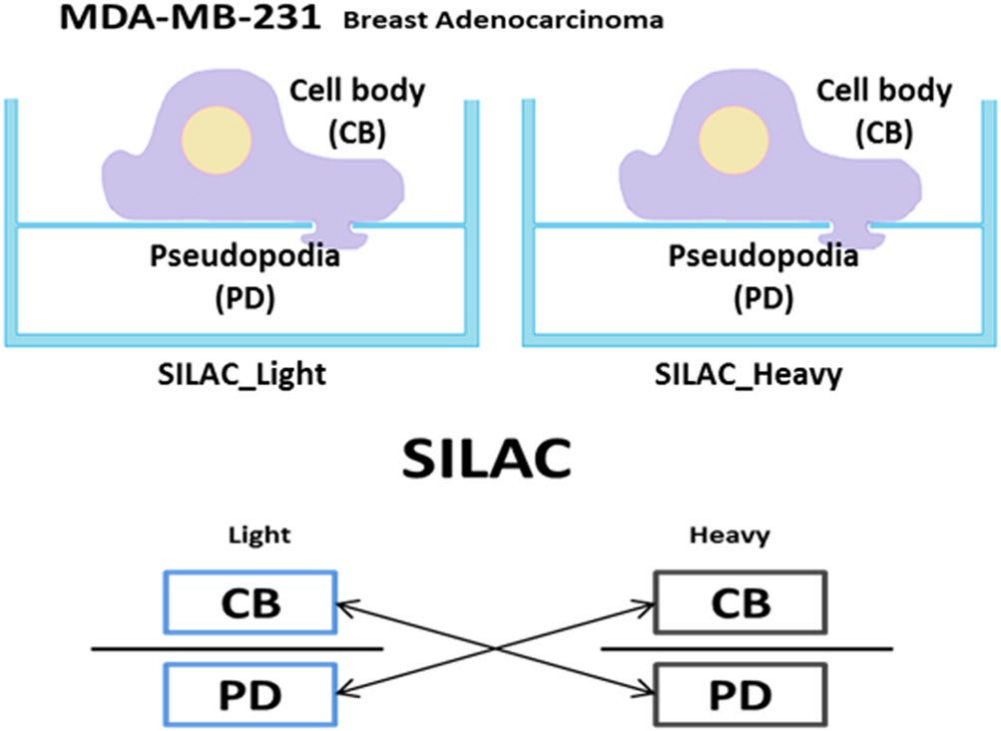
Quantitative proteomics profiling strategy for pseudopodia of MDA-MB-231 cells using SILAC. Illustration of MDA-MB-231 cell extending pseudopodia. Cells are cultured in SILAC MDEM and extend pseudopodia through 3-μm pores of membrane filter in response to serum stimulation (up). Pseudopodia are separated from cell bodies manually. The isolated cell bodies and pseudopodia are mixed 1:1 in a label swapping scheme and then profiled using quantitative proteomics (bottom).

Figure 2 shows the strategy employed for quantitative pseudopodia proteomics in MDA-MB-231 cells. Purification of pseudopodia and cell bodies, was achieved through a previously described protocol based again on microporous transwell filters to separate the structures^14,15^. In order to perform quantitative proteomic analysis of pseudopodia, we cultured MDA-MB-231 cells using stable isotope labeling by amino acids in cell culture^16,17^ for in excess of 6 cell divisions and then isolated pseudopodia and cell body fractions after serum stimulation for 2 h. To check incorporation rate of heavy labeled Arginine and Lysine, we confirmed heavy labeling peptides in cell lysates in heavy SILAC by LC/MS/MS (Supplementary Fig. 2A) and measured the ratio of heavy labeled peptides to all peptides identified. The overall incorporation rate was approximately 99%, reflecting the enrichment of the reagents used (Supplementary Fig. 2B). For SILAC-based comparative proteomic analysis between pseudopodia and cell bodies, we employed a strategy where light and heavy labeled cell body samples were combined with heavy and light labeled pseudopodia samples, respectively, in a label swapping mixing scheme.

By comparing SILAC labeling ratios between pseudopodia and cell body, we identified 578 proteins to be enriched in pseudopodia (Supplementary data), including known pseudopodial proteins such as calpain, filamin, IGQAP1, β1 integrin, and ROCK^18^, which validate the approach. The small number of cell body-deriving proteins, contained in the data set may stem from leaking of highly abundant proteins during the extraction procedure, which in conjunction with a comparatively low protein concentration in the pseudopodia fraction may lead to an apparent over-representation. We proceeded with annotation of pseudopodia enriched proteins using gene ontology (GO) analysis including GO Biological process, GO molecular function, GO cellular component^19^, and KEGG pathways^20^ (Supplementary data). Enriched GO categories include cytoskeleton, plasma membrane, mitochondria and cytosol. Enriched KEGG pathways include regulation of actin cytoskeleton, endocytosis, proteasome, GAP junction, and glycolysis. Groups with log_2_ ratios in excess of 3 (pseudopodia vs. cells bodies) were associated with GAP junction, endocytosis, tight junction, and glycolysis, while log_2_ ratios between 3 and 1 were mainly involved in regulation of actin cytoskeleton and the ubiquitin/proteasome system. Among the proteins identified as enriched in pseudopodia were, IQGAP1 and ARPC (Actin related protein complex). IQGAP1, a key regulator of adhesion and migration, is known to regulate an array of molecular events, such as N-Wasp induced branched actin assembly by ARPC^21^. Further pseudopodia-enriched proteins are ILK1, PARVA (Alpha-parvin) and RSU (Ras suppressor protein 1), which are known to cooperate in promotion of metastasis. ILK1 is a serine/threonine protein kinase which interacts with integrin subunits to regulate integrin mediated signal transduction^22^. It is upregulated in several cancer types^23,24^ and was accordingly interrogated for influence on cancer cell migration below.

**Figure 3:**
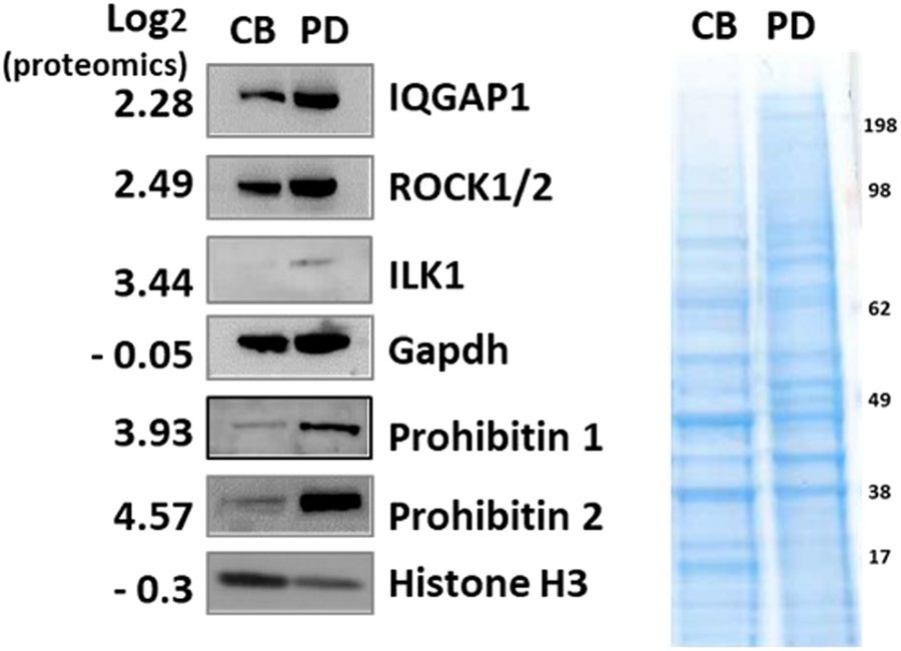
Validation of results from the quantitative proteomics screen using Western Blot. Western Blots were performed for proteins from MDA-MB-231 cell pseudopodia (PD) and cell body samples (CB; left). Equal amounts of protein samples were separated by SDS PAGE, plotted and probed with antibodies specific for the indicated proteins, as well as stained with Coomasie Blue (right).

To validate the quantitative proteomic analysis of pseudopodia, a subset of proteins enriched in pseudopodia or cell body fractions were examined by Western blotting (Fig. 3) and showed good correlations with the proteomics results. Histone proteins were present mostly in the cell body fraction, and IQGAP1 and ROCK1/2 were present in the pseudopodia fraction.

Table 1 shows the top 50 proteins identified as highly enriched in the pseudopodia fraction. Interestingly, this includes a number of mitochondrial proteins like SLC25A, LRPPRC, GOT2, ATP5A, ACADM and PHBs (Prohibitions). PHBs proteins are evolutionarily conserved and widely expressed in diverse cellular processes such as proliferation and metabolism, are known to interact with various receptors, and are involved with activation of ERK signaling in the plasma membrane. They localize to the mitochondrial inner membrane^25–27^.

**Table 1:** List of the top 50 proteins identified as highly enriched in pseudopodia over cell bodies.

**Gene_symbol**	**Protein_names**	**Log**_**2**_ **ratio**	***p_values***	***FDR*** ^*^ ***_values***
SLC25A6	ADP/ATP translocase 3	5.40	1.53E-05	3.51E-04
TBB2	Tubulin beta-2 chain	5.05	2.87E-07	1.93E-04
GOT2	Aspartate aminotransferase, mitochondrial	4.97	2.46E-05	4.25E-04
PHB2	Prohibitin-2	4.58	7.21E-05	7.15E-04
SLC25A1	Tricarboxylate transport protein, mitochondrial	4.50	1.48E-04	1.05E-03
SLC25A24	Calcium-binding mitochondrial carrier protein SCaMC-1	4.46	9.19E-04	3.49E-03
ATP5A1	ATP synthase subunit alpha, mitochondrial	4.37	9.73E-04	3.60E-03
ARHGAP1	Rho GTPase-activating protein 1	4.34	6.93E-04	2.82E-03
TUBB3	Tubulin beta-3 chain	4.13	1.25E-04	9.64E-04
TUBB8	Tubulin beta-8 chain	4.05	1.12E-04	8.94E-04
WDR1	WD repeat-containing protein 1	4.02	7.00E-06	2.65E-04
GDI2	Rab GDP dissociation inhibitor beta	3.97	4.86E-06	2.55E-04
PHB	Prohibitin	3.94	6.01E-04	2.56E-03
Eif3e	Eukaryotic translation initiation factor 3 subunit E	3.88	2.21E-04	1.32E-03
CCT7	T-complex protein 1 subunit eta	3.84	3.90E-05	5.02E-04
TUBB	Tubulin beta chain	3.81	6.30E-04	2.64E-03
TUBA3	Tubulin alpha-3 chain;Tubulin alpha-5 chain	3.81	3.38E-03	8.70E-03
FH	Fumarate hydratase, mitochondrial	3.80	1.63E-04	1.11E-03
EHD4	EH domain-containing protein 4	3.80	4.61E-04	2.15E-03
ETF1	Eukaryotic peptide chain release factor subunit 1	3.77	7.09E-05	7.12E-04
UMPS	Uridine 5-monophosphate synthase;Orotate phosphoribosyltransferase	3.73	1.29E-04	9.75E-04
SLC25A3	Phosphate carrier protein, mitochondrial	3.72	2.01E-04	1.25E-03
ACTR10	Actin-related protein 10	3.71	2.07E-03	6.07E-03
TUBB2B	Tubulin beta-2B chain	3.66	2.08E-06	2.21E-04
EPHX1	Epoxide hydrolase 1	3.62	1.29E-04	9.75E-04
NDA3	Tubulin beta chain	3.61	1.03E-03	3.74E-03
SLC16A3	Monocarboxylate transporter 4	3.60	1.87E-06	2.21E-04
SLC7A5	Large neutral amino acids transporter small subunit 1	3.60	1.68E-05	3.72E-04
TUBG1	Tubulin gamma-1 chain;Tubulin gamma-2 chain	3.56	8.38E-04	3.25E-03
ACADM	Medium-chain specific acyl-CoA dehydrogenase, mitochondrial	3.53	4.04E-04	1.99E-03
PICALM	Phosphatidylinositol-binding clathrin assembly protein	3.53	3.38E-03	8.70E-03
ATP1B1	Sodium/potassium-transporting ATPase subunit beta-1	3.52	1.24E-05	3.22E-04
PAICS	Multifunctional protein ADE2	3.50	1.08E-03	3.87E-03
NXN	Nucleoredoxin	3.48	5.29E-05	6.02E-04
PSMD12	26 S proteasome non-ATPase regulatory subunit 12	3.48	6.91E-04	2.82E-03
SLC3A2	4F2 cell-surface antigen heavy chain	3.47	7.52E-05	7.34E-04
CORO1C	Coronin-1C	3.47	1.78E-03	5.44E-03
OXSR1	Serine/threonine-protein kinase OSR1	3.45	1.37E-04	1.01E-03
CCT	T-complex protein 1 subunit gamma	3.45	1.25E-06	2.21E-04
GPI	Glucose-6-phosphate isomerase	3.45	4.87E-04	2.23E-03
ILK1	Integrin-linked protein kinase1	3.45	5.62E-04	2.44E-03
PSMC3	26 S protease regulatory subunit 6 A	3.44	3.42E-05	4.84E-04
PNP	Purine nucleoside phosphorylase	3.42	1.83E-05	3.84E-04
HSD17B11	Estradiol 17-beta-dehydrogenase 11	3.41	1.47E-05	3.43E-04
EHD1	EH domain-containing protein 1	3.39	1.94E-04	1.23E-03
FERMT2	Fermitin family homolog 2	3.38	2.29E-03	6.45E-03
G6PD	Glucose-6-phosphate 1-dehydrogenase	3.35	3.52E-04	1.84E-03
SLC1A5	Neutral amino acid transporter B	3.33	1.76E-04	1.15E-03
CS	Citrate synthase, mitochondrial	3.33	1.91E-03	5.76E-03
ASNS	Asparagine synthetase [glutamine-hydrolyzing]	3.32	2.03E-04	1.25E-03

^*^FDR: False discovery rate (corrected p-value according to Benjamini-Hochberg^1^).

To exclude the possibility that recovery of mitochondrial proteins in the pseudopodia fraction was artifactual, we checked whether mitochondria localize to pseudopodia using phalloidin (green) and mitotracker (red) for staining of F-actin and mitochondria, respectively, in transwell culture. Although the majority of mitochondria were present in the cell body, they also show a clear presence in the pseudopodia (Supplementary Fig. 3). This finding is further supported by a recent report on a pseudopodia-subtype involved in myelination of Schwann cells, which also describes PHBs and mitochondria to be present in pseudopodia – with functional implications for myelination^28^. We conclude that mitochondria and in particular PHBs are likely components of pseudopodia in cancer cells.

### PHBs are Necessary for Cancer Migration *in vitro*

**Figure 4:**
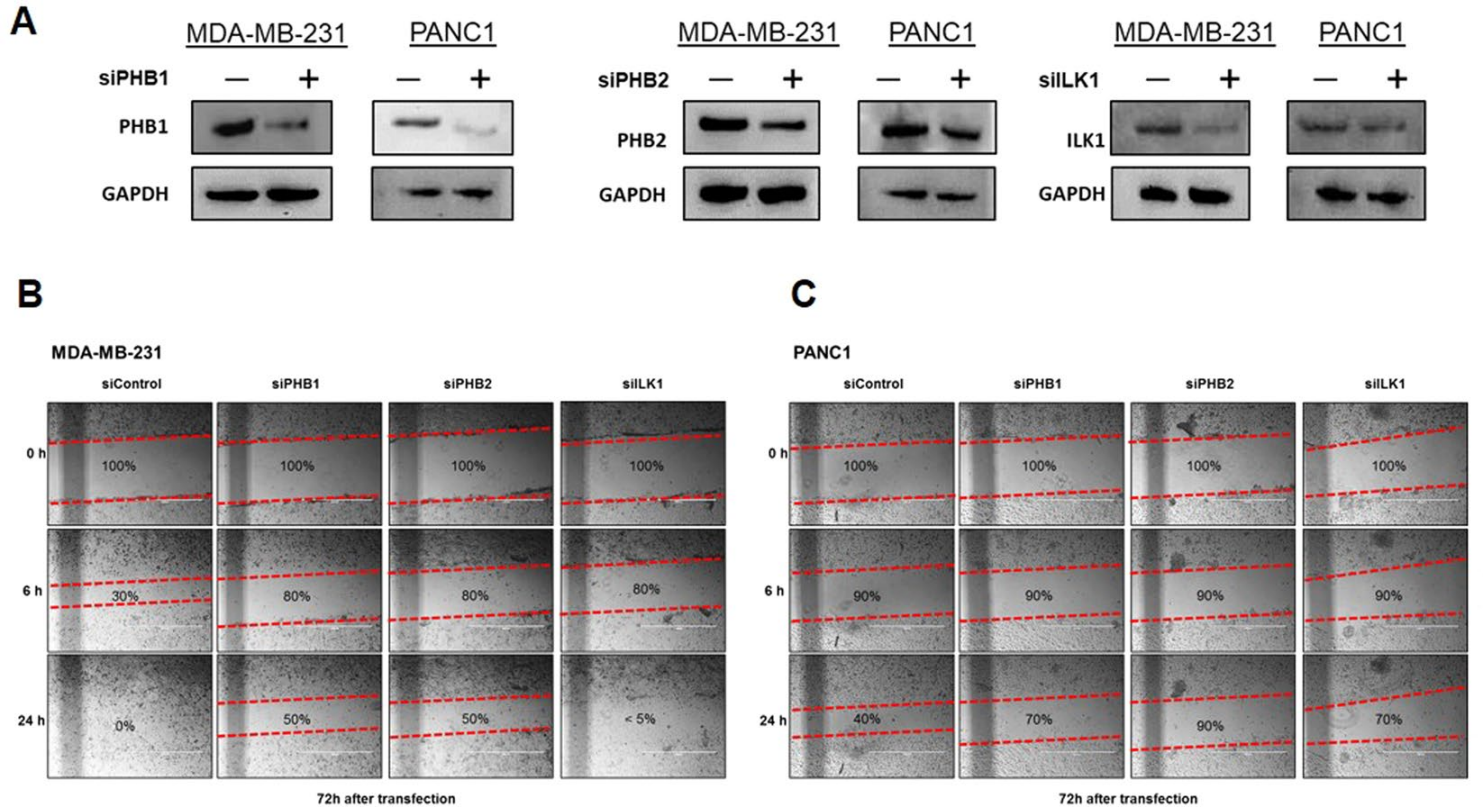
Wound healing assay of siRNA knockdown in MDA-MB-231 and PANC1 cells. (**A**) Western Blot demonstrating knockdown of PHB1, PHB2, and ILK1 in MDA-MB-231 and PANC1 cells. (**B** and **C**) siRNA knockdown of PHBs or ILK1 inhibits migration of different types of cancer cells. Each gap was created in confluent cells transfected with siRNA against PHBs or ILK1 and the evolution of the gap width was measured over time. Scale bar = 200 μm.

The role of pseudopodia in cell migration is well established. We thus hypothesized that disrupting a key player in pseudopodia physiology impacts migration. To explore the relevance of our candidate proteins in this context, we used two different invasive cancer cell lines. MDA-MB-231 and PANC1, models for breast and pancreatic cancer, respectively, were transfected with siRNA knocking down PHB1 and PHB2. In addition, expression of ILK1, which was also found enriched in pseudopodia above, was knocked down by siRNA in both cell lines. Reduction of protein expression by siRNA is shown for all experiments by western blot in Fig. 4A. To check the effect of knockdown PHB1, PHB2 and ILK1 on cell proliferation, we performed cell proliferation assay, observing slightly reduced proliferation in all cases (Supplementary Fig. 4A). Using a wound healing assay, we found that the PHB1 and PHB2 knockdown in both MDA-MB-231 (Fig. 4B) and PANC1 (Fig. 4C) had a reduced propensity for migration and gap closure when compared to control cells expressing a Non-targeting siRNA. Interestingly, migration in cells with ILK1 knockdown, was impacted only for PANC1, while MDA-MB-231 showed very little difference in migration pattern or rate.

### Rocaglamide Inhibits Directional Migration of Cancer Cells

**Figure 5:**
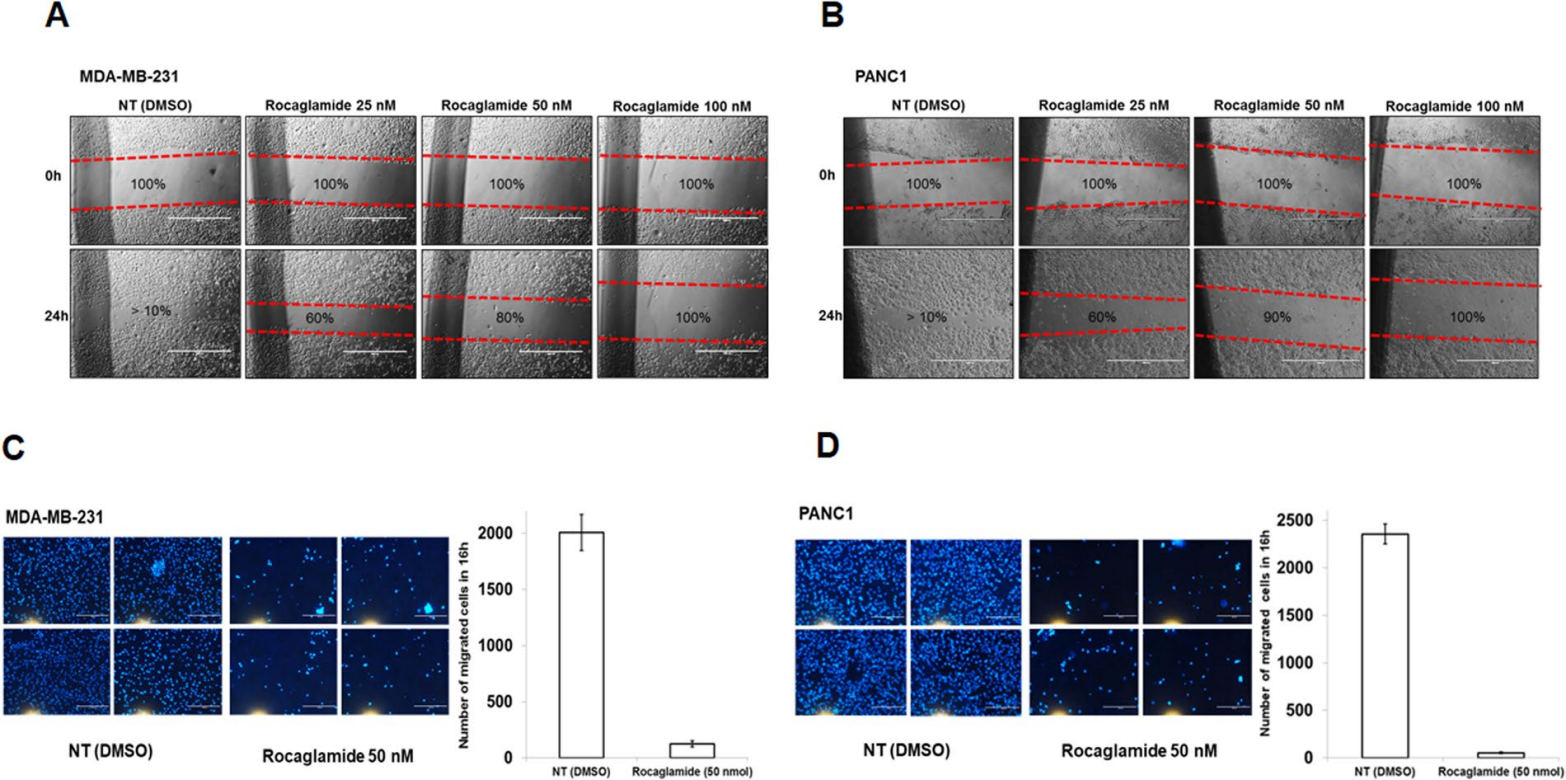
Wound healing assay and directional migration assay of Roc-A treated MDA-MB-231 and PANC1 cells. (**A** and **B**) Roc-A inhibits migration of cancer cells. MDA-MB-231 and PANC1 cells were treated without (control) or with Roc-A (25 to 100 nM) and the evolution of gap closure was measured. (**C** and **D**) Roc-A inhibits directional migration of cancer cells. MDA-MB-231 and PANC1 cells treated without (control) or with 50 nM Roc-A for 24 h were subjected to a transwell migration assay in response to serum stimulation. Scale bar = 200 μm.

Rocaglamide (Roc-A), a naturally occurring flavagline^29,30^, is reported to inhibit the RAF-MEK-ERK pathway by targeting PHB1 and PHB2^31^. Given our findings above, we therefore sought to determine its effect on cancer cell migration and repeated the wound healing assay described above in the presence of increasing doses (25 to 100 nM) of Roc-A. In alignment with the knockdown data described above, we found Roc-A to reduce migration of MDA-MB-231 (Fig. 5A) and PANC1 (Fig. 5B) cells after 24 h of treatment even at low concentrations (25 nM). Translating this finding to an alternative experimental system, we performed a directional cell migration assay. Migration of MDA-MB-231 (Fig. 5C) and PANC1 (Fig. 5D) cells into the bottom chamber of a transwell assembly was strongly stimulated by serum, while cells treated additionally with Roc-A barely moved, in spite of serum stimulation. To check the effect of Roc-A on cell proliferation, we repeated the cell proliferation assay in its presence. While we observed a dose dependent proliferative effect of Roc-A with significantly reduced proliferation at 100 nM final concentration, the effect at 25 nM and 50 nM used in the assays above was mild, supporting interpretation of our observations as a migration, rather than proliferative phenotype (Supplementary Fig. 4B).

## Discussion

Metastasis is the major cause of mortality in cancer-affected patients and there is currently no generic treatment for patients suffering from it. Therefore, development of new anticancer drugs that efficiently tackle metastasis is crucial.

Cancer cell migration is the essential first step for invasion and metastasis formation. Using SILAC-based quantitative proteomics in combination with physical pseudopodia fractionation in MDA-MB-231 cancer cells, we devise in this study a strategy to identify proteins that may be involved with cell migration and thus serve as potential anti-metastatic targets for therapeutic purposes. Our results provide robust and reliable data for investigation of cancer metastasis research.

In the top 50 proteins most enriched in pseudopodia over cell bodies we found, as expected, many factors involved in cell adhesion, migration, and metabolism. As numerous studies suggest that cancer cell metabolism, such as the switch from oxidative metabolism to glycolysis, plays an important part in tumor cell migration, invasion, as well as growth^32–37^ and it was also reported that localization of mitochondria within moving cells have strong correlations with the speed of its movement as well as directional persistence during directional migration^38^, the inclusion of a large number of mitochondrial proteins in the enriched set may not be as surprising as it initially appears.

Pseudopodia are large dynamic actin cytoskeleton based protrusions formed at the cell front and intimately linked to directional migration. The implied high level of energy consumption may require redistribution of mitochondria to pseudopodia in moving cancer cells for directional migration and thus possibly increased invasiveness. Future studies of the mitochondrial localization may thus contribute to a better understanding of the mechanism of cancer invasion, migration and finally cancer metastasis.

The pseudopodia/mitochondria connection hypothesized is exemplarized by our finding that PHBs were significantly enriched in pseudopodia of cancer cells. Multiple studies have reported PHBs as predominantly localized to mitochondria, and playing a key role in maintenance of mitochondrial function and morphology^39,40^. Our follow-up experiments demonstrate that depletion of PHBs strongly reduces cancer cell migration in general and directional migration more specifically-even in the context of serum stimulation.

Our findings are supported by a similar study published recently describing PHBs as enriched in pseudopodia of Schwann cells with possible important implications for central nervous system myelination^28^.

Accordingly, we assume that accumulation of PHBs in pseudopodia is mechanistically linked to cell migration in general and cancer cell migration more specifically.

We proceeded with exploiting this hypothesis pharmacologically and show that Roc-A, a natural anticancer compound known to bind to PHB1 and PHB2 directly^31^, indeed appears to interfere with cancer cell migration in a wound healing and directional migration assay, both, even at low concentrations (25 to 100 nM). Replicating our results in cellular models deriving from different cancers (MDA-MB-231 from breast cancer and PANC1 from pancreatic cancer) hints at a general, rather than cell type-specific nature of the observed effect. We thus believe Roc-A to be an interesting candidate drug for anti-cancer metastatic activity in cancer in general.

In summary, we present a quantitative proteomics strategy for the identification of factors involved in cancer cell motility and metastasis and utilize it to identify PHBs as proteins likely involved in the process. This in turn leads us to hypothesize that localized metabolism supported by mitochondrial relocalization may be a vital factor in function of pseudopodia. We further extend our findings to describe Roc-A, a PHB directed compound, as an inhibitor of cancer cell migration, leading us to conclude that it may represent a promising drug candidate targeting metastasis reduction or prevention.

While this study focused on the effects of PHBs and a single PHB inhibitor, we believe that other proteins characterized by us as enriched in pseudopodia may provide further insight into the process of cancer cell migration, invasion and metastasis, as well as serving as targets for further pharmacological interference with those processes. For instance, our result show that ILK1 knockdown in PANC1 cells reduced migration capacity. As an agent called compound 22 (cpd 22) is an ILK1 inhibitor already described in the literature^41^, this target/compound pairing may warrant future exploration in at least a subset of cancers. Similarly, the vacuolar protein sorting-associated protein (VPS) family and STAT proteins may be inhibited by Bafilomycin^42^ and Nifuroxazide, respectively^43^. Our proteomic analysis of pseudopodia of cancer cells may thus offer promising avenues for the development of strategies to combat metastatic disease.

## Methods

### Pseudopodia Assay

MDA-MB-231 human breast cancer cells were starved for 16 h, subsequently plated into the top Boyden chamber of polycarbonate transwell inserts with 3-μm pores (Corning #3414; 1.5 × 10^6^ cells), and incubated for 2 h at 37 °C. To stimulate formation of pseudopodia from the cell surface, the inserts were transferred to wells containing serum-free Dulbecco’s modified Eagle’s medium (DMEM) (HyClone, Thermo Scientific) (no stimulus) and DMEM with 100 ng/mL lysophosphatidic acid (LPA), or DMEM with 1% fetal bovine serum (FBS), respectively and incubated for 2 h at 37 °C. We also tested 0.1% and 0.5% of serum stimulation (Supplementary Fig. 1). Cell bodies in the top chamber and pseudopodia in the bottom chamber were stained using phalloidin (DyLight 488 nm, #21833, Thermo Scientific) after three PBS washes and fixing with 4% paraformaldehyde.

### SILAC Based Cell Culture and Pseudopodia Extraction for Quantitative Proteomics

MDA-MB-231 cells were grown in SILAC DMEM (#88425, Thermo Scientific) containing natural lysine and arginine in the ‘light’ or ^13^C_6_^15^N_2_-lysine (Lys8, #CNLM-291, (^13^C_6_, 99%; ^15^N_2_, 99%), Cambridge Isotope laboratory Inc) and ^13^C_6_^15^N_4_-arginine (Arg10, #CNLM539, (^13^C_6_, 99%; ^15^N_4_, 99%), Cambridge Isotope laboratory Inc) in the ‘heavy’ condition, along with antibiotics and dialyzed FBS. For complete labeling, cells were cultured in excess of 6 doublings in SILAC DMEM and an incorporation test was performed to measure the ratio of heavy labeled peptides to all peptides identified (heavy and light). In order to prepare a pseudopodia fraction, we used the specific enrichment method described in Klemke *et al*.^15^. Protein yield was measured using a BCA assay. Twenty micrograms of cell body sample from the top and pseudopodia sample from the bottom chamber were prepared, respectively, and for comparative proteome profiling mixed 1:1 in a label swapping scheme.

### Proteomics Analysis

For LC/MS/MS analysis, a tryptic digest of each sample was injected using a nano-flow chromatographic system (Easy nLC-II, Thermo Fisher Scientific, Bremen, Germany) and separated on in-house packed column emitters using a 20 cm fused silica capillary (i.d. 75 μm, o.d. 360 μm) filled with C18 (particle size 3 μm, 100 Å) resin (Dr. Maisch GmbH, Ammerbuch-Entrigen, Germany). Separation of peptides was performed by a 120 min gradient from buffer A (0.5% acetic acid in water) to buffer B (80% acetonitrile, 0.5% acetic acid). Eluted peptides were electrospray ionized and introduced into a Q Exactive mass spectrometer. Precursor ion scans were acquired at a resolution of 70,000 (*m/z* 300). A data dependent analysis was used, where the 10 most intense ions detected in the full scan are isolated (3 Th isolation width) and fragmented using higher energy collisional dissociation (HCD, collision energy 25) in the context of dynamic exclusion for 25 s. Unassigned charge states and singly charged ions were also excluded.

Protein identification and quantification were performed using the MaxQuant suite of algorithms (version 1.4.1.2)^44^ and a *Homo sapiens* UniprotKB data base (downloaded on 04.06.2015;109,411 protein entries). The search parameters employed are as follows: first search mass accuracy tolerance 20 ppm, main search mass accuracy tolerance 4.5 ppm, FTMS MS/MS tolerance 20 ppm, minimum peptide length of 7 amino acids, peptide spectrum match false discovery rate (FDR) and protein FDR both set to 0.01 as calculated by the reverse database approach^45,46^. Carbamidomethylation of cysteine was searched as a fixed modification, and N-terminal acethylation and oxidation of methionine were searched as variable modifications. Heavy labeled arginine (Arg10) and lysine (Lys8) were specified for quantitative analysis.

The mass spectrometry proteomics data have been deposited to the ProteomeXchange Consortium via the PRIDE^47^ partner repository with the dataset identifier PXD004658.

### Western Blotting for Candidate Validation

To validate proteomics results, western blot analysis was performed. After the transfer of target proteins from SDS-PAGE, blocked membranes were incubated with Millipore anti-ROCK2 (Rho-associated protein kinase 2) (#07-1458), anti- IQGAP1 (Ras GTPase-activating-like protein 1) (#abt186), anti-PHB1 (#abn293), anti-PHB2 (#mabc953); Cell Signaling anti-GAPDH (#2118), anti-ILK1 (Integrin-linked protein kinase 1) (#3862), and Anti-Histone H3 (#4499) overnight at 4 °C. The membrane was rinsed with 1x TBS buffer prior to incubation with secondary antibody for 1 h. Blots were developed using ECL buffer (GE Healthcare) and the Geliance 600 imaging system (PerkinElmer).

### Bioinformatical and Statistical Analyses

All statistical analysis of MaxQuant results was performed by autonomics, an in-house suite for the statistical analysis of omics data (A.M. Bhagwat, R.J. Cotton, L. Cougnaud, and J. Graumann, manuscript in preparation; https://bitbucket.org/graumannlabtools/autonomics). The experimental design consisted of 4 replicates for both pseudopodia as well as cell body. MaxQuant was used to transform the raw spectra into a matrix of protein group ration (row = protein group, column = sample). From this matrix, we retained proteins which were not tagged by MaxQuant as contaminants or reverse hits, and which had at least two ratios associated. We subsequently log_2_ transformed the ratios to obtain approximately symmetric distributions. Log_2_ protein ratios were quantile normalized prior to statistical analysis^48^. For statistical analysis, the model “protein ratio ~ 1” was fitted in parallel for each protein using the R^49^ package limma^48^, For each protein group, this model tests whether the log_2_ ratio is significantly different from zero, using limma’s built-in moderated t-test. Benjamini-Hochberg FDR values were calculated using the p.adjust function in R^50,51^.

### siRNA Knockdown of Protein Expression and Wound Healing Assay

The MDA-MB-231 human breast cancer cell line and PANC1 human pancreatic cancer cell line were transiently transfected using siRNA against PHB (siGENOME Human PHB (5254) siRNA – SMART pool; Dharmacom), PHB2 (siGENOME Human Phb2 (11331) siRNA – SMART pool; Dharmacon), ILK1 (siGENOME Human Ilk (3611) siRNA – SMART pool; Dharmacon), and control (siGENOME Non-Targeting siRNA Pool; Dharmacon) using DharmaFECT1 transfection reagent (#T-2001-02; Dharmacon) for 72 h. For validation of the transfection efficiency, western blot analysis was employed. In a wound healing assay, cells were plated in 12-well plates (#3043, Becton Dickinson) and cultured. After transfection, a narrow gap was created with a pipette tip in the confluent cell monolayer and monitored for 24 h for recolonization.

### Treatment with Rocaglamides and Wound Healing/Migration Assay

MDA-MB-231 and PANC1 cells were cultured in DMEM with 10% FBS until 90–100% confluence in 12-well plates (#3043, Becton Dickinson) and subsequently treated with DMSO (control), 25 nM, 50 nM, and 100 nM of Roc-A (Rocaglamides, #84573-16-0 ≥96% pure, Sigma Aldrich) dissolved in DMSO. Wound healing was assayed for 24 h as described above.

For the migration assay, polycarbonate transwell inserts with 8-μm pores (Corning #3422) were used. MDA-MB-231 and PANC1 cells were cultured in DMEM with 10% FBS and subsequently starved for 16 h. Trypsinized cells were plated into the top Boyden chamber inserts and incubated for 2 h at 37 °C. The inserts were transferred to a plate containing DMEM with 1% FBS and incubated overnight at 37 °C. Subsequently cell presence in top and bottom chambers was evaluated using DAPI (#p36935, molecular probes by Life Technologies).

### Cellular Proliferation Assay

For cellular proliferation assay, Cell Proliferation ELISA, BrdU (colorimetric) Version 14.0 (#11647229001, Roche Applied Science, Mannheim, Germany) was used. Cells were labeled with BrdU labeling solution for 2 h at 37 °C. After fixing the labeled cells, cells were labeled with Anti-BrdU-POD working solution for 90 min. Cells were washed with Washing solution and then incubated with Substrate solution for 15 min. BrdU assay was performed by measuring the absorbance at 450 nm. All tests were conducted in triplicates and repeated twice.

## Electronic supplementary material

Supplementary Information

Supplementary Dataset GOBP

Supplementary Dataset GOCC

Supplementary Dataset KEGG

Supplementary Dataset GOMF

Supplementary Dataset Pseudopodia protein ID
