# Supplementary material for: Proteomic profiling of human cancer pseudopodia for the identification of anti-metastatic drug candidates
Source: Sci Rep. 2018 Apr 11;8:5858. doi: 10.1038/s41598-018-24256-8 (PMC5895739; doi:10.1038/s41598-018-24256-8)
Supplement: Supplementary file 1 — Supplementary Information [file 41598_2018_24256_MOESM1_ESM.docx]

**Proteomic profiling of human cancer pseudopodia for the identification of anti-metastatic drug candidates**

**Sunkyu Choi**^1^, **Aditya M. Bhagwat**^1^, **Rasha Al Mismar**^1^, **Neha Goswami**^1^, **Hisham Ben Hamidane**^1^, **Lu Sun**^1^, & **Johannes Graumann**^1,2^

^1^Research Division, Weill Cornell Medicine - Qatar, Doha, State of Qatar

^2^Current address: Scientific Service Group Biomolecular Mass Spectrometry, Max Planck Institute for Heart and Lung Research, Bad Nauheim, Germany

^*^ To whom correspondence should be addressed: Johannes.Graumann@mpi-bn.mpg.de

**Supplementary figure 1**


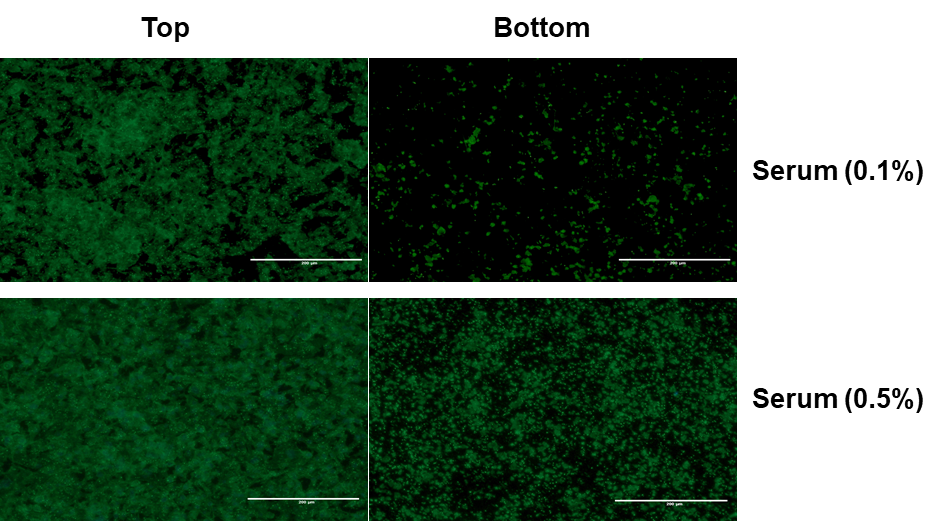


**Extention of pseudopodia by MDA-MB-231 cells in response to 0.1% and 0.5% serum.** Microscope images of MDA-MB-231 cell bodies (top) or pseudopodia (bottom) in response to 0.1% and 0.5% serum. Cells were stained for F-actin with phalloidin. Scale bar = 200 μm.

**Supplementary figure 2**


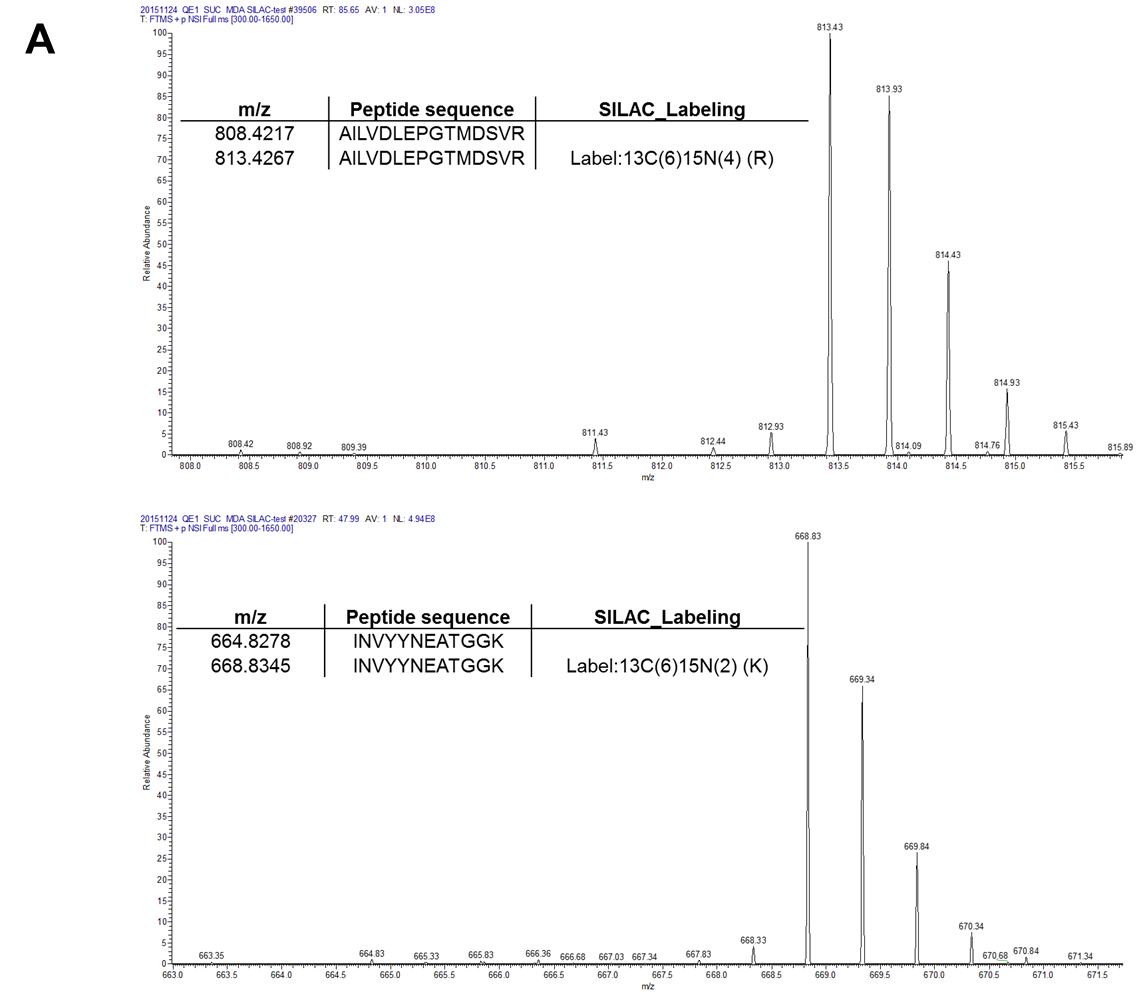


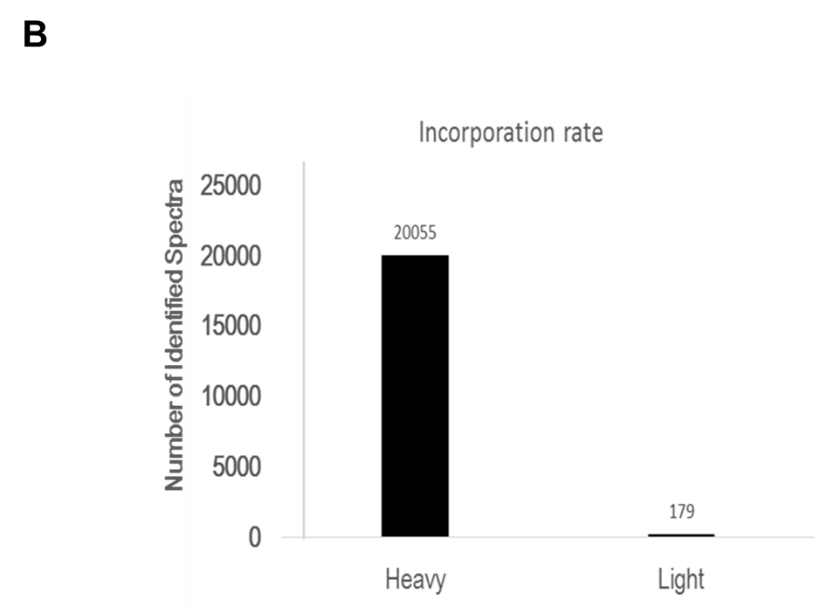


**SILAC quality controls.** (A) MS spectra showing peptides in heavy SILAC. (B) Incorporation rate of heavy labeling for SILAC analysis. The incorporation test was performed measuring the ratio of peptides identified as heavy labeled to all identified peptides.

**Supplementary figure 3**


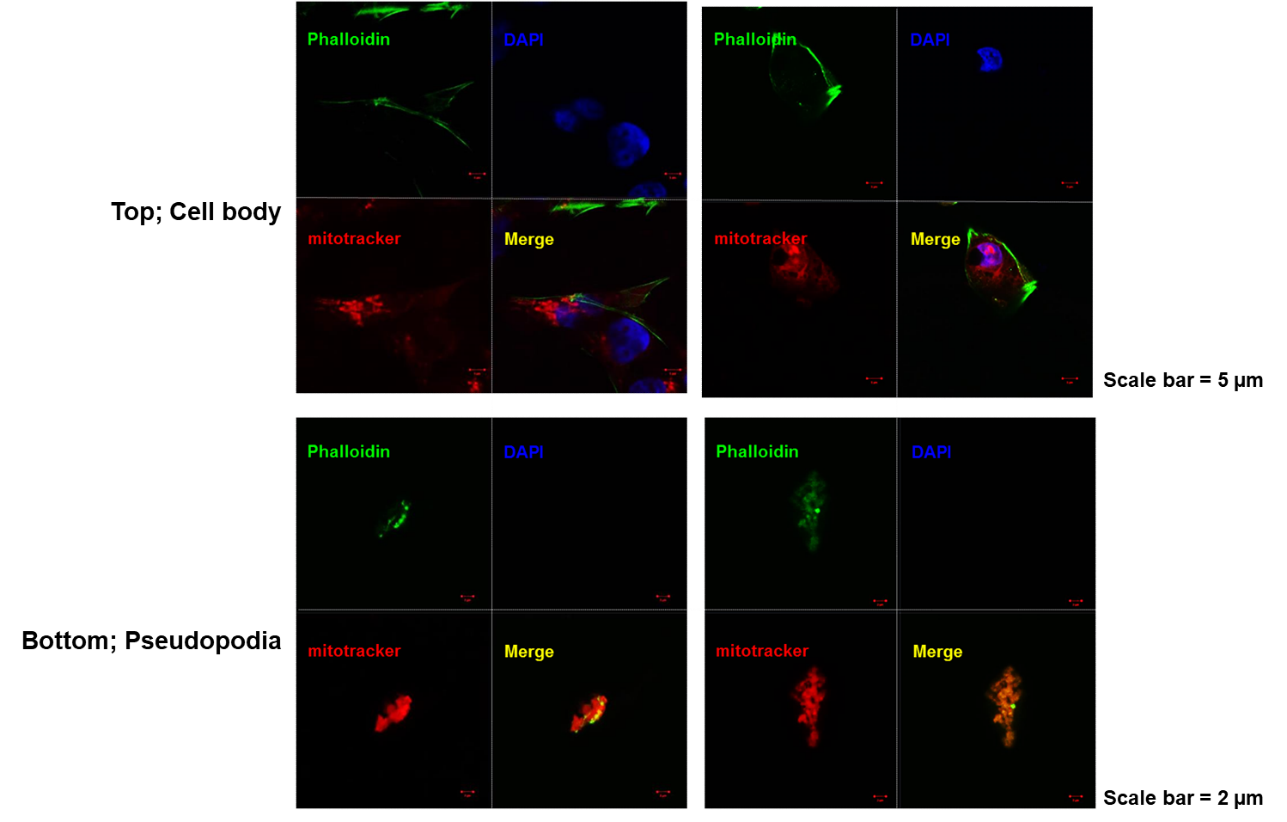


**Pseudopodia contain mitochondria.** Mitotracker staining (red) in cells on the top chamber (cell body) and bottom chamber (pseudopodia). F-actin proteins were stained with phalloidin (green) and nuclei were stained using DAPI (blue).

**Supplementary figure 4**


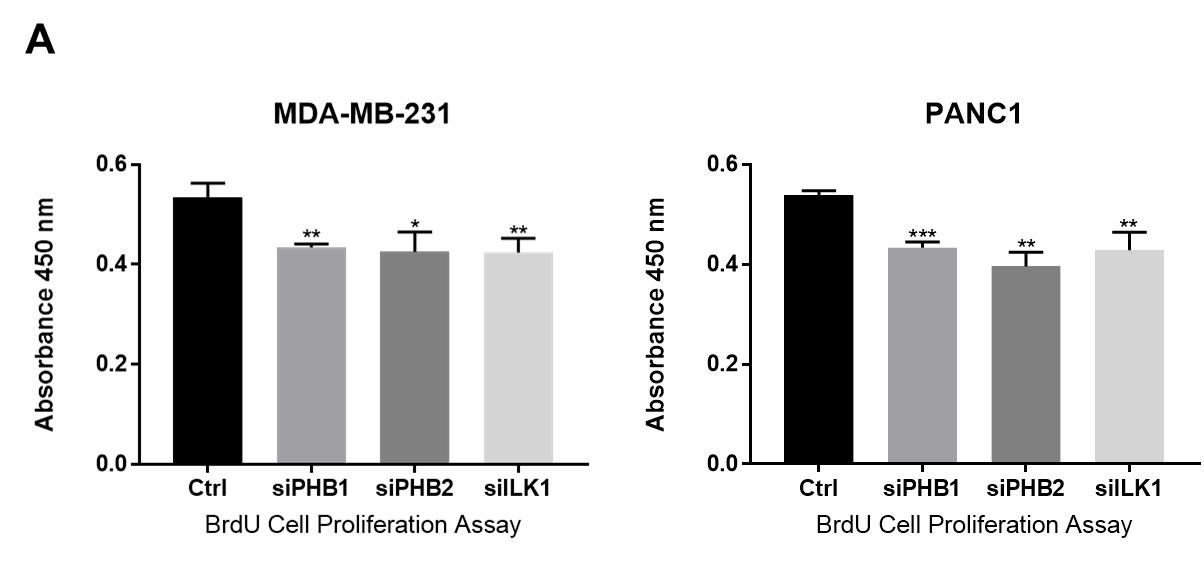


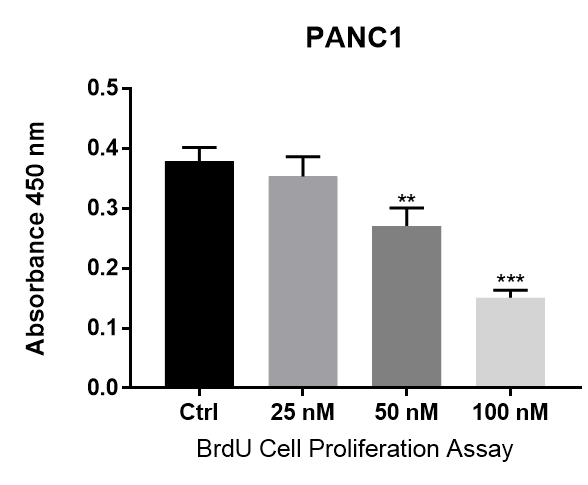

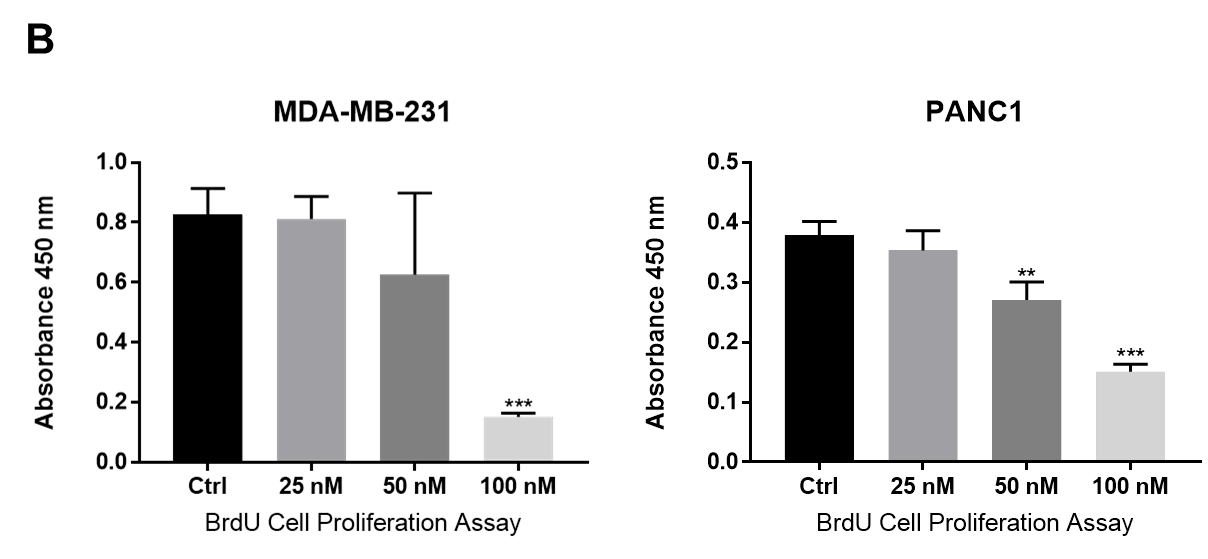


**B**

**Cell proliferation analysis using BrdU assay.** (A) BrdU assay of the effects of knockdown PHB1, PHB2 and ILK1 on cell proliferation. (B) BrdU assay of the effects of Roc-A treatments on cell proliferation. BrdU labeling was performed as described in Methods and measured by 450 nm absorbance. All tests were conducted in triplicates and repeated twice. Error bars are shown. **P < 0.05*, ***P < 0.01* and ****P < 0.001*.

**Original pictures of the Western Blots shown in the Figure 3.**

**
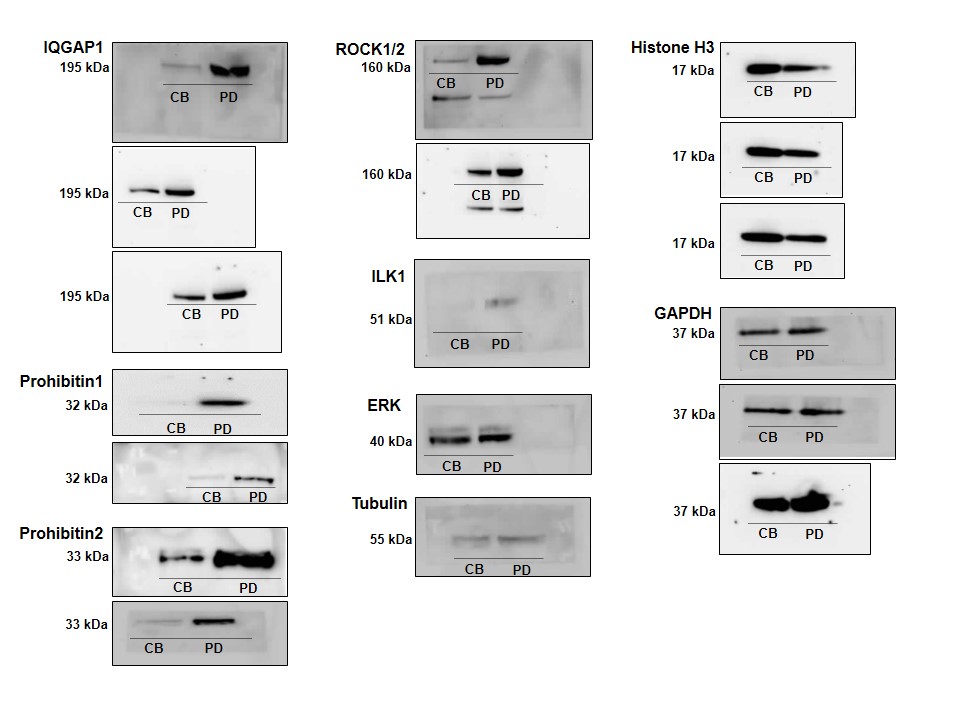
**

CB : Cell body, PD : Pseudopodia

**Original pictures of the Western Blots shown in the Figure 4A**

**
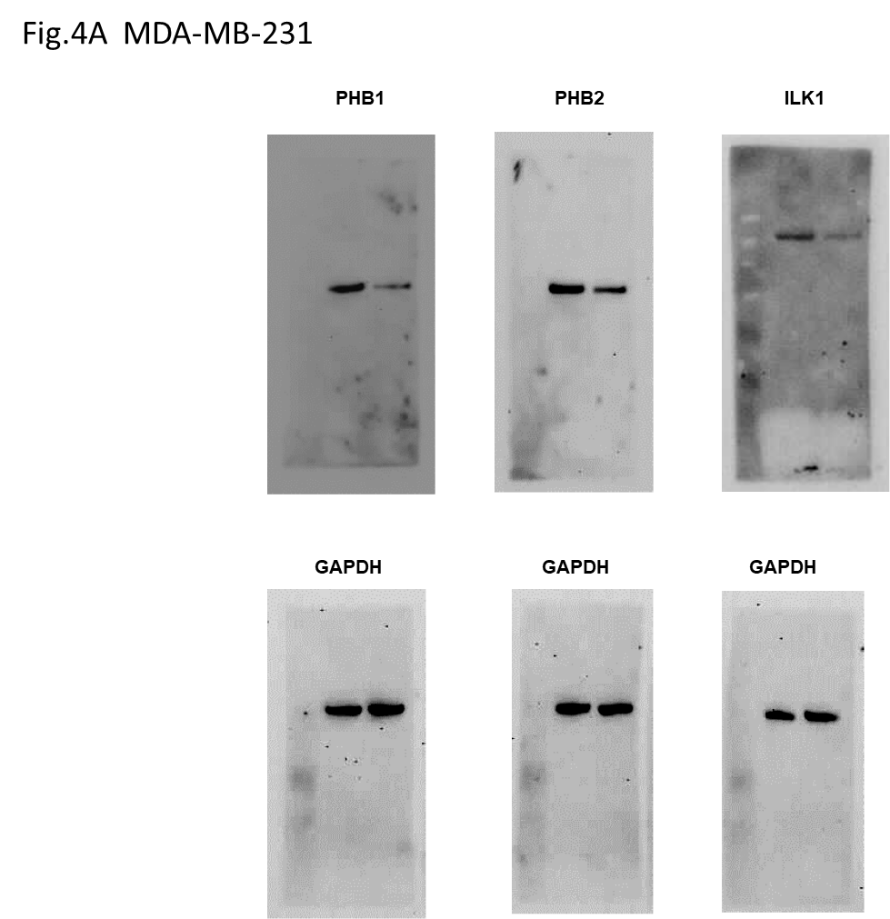
**

**.
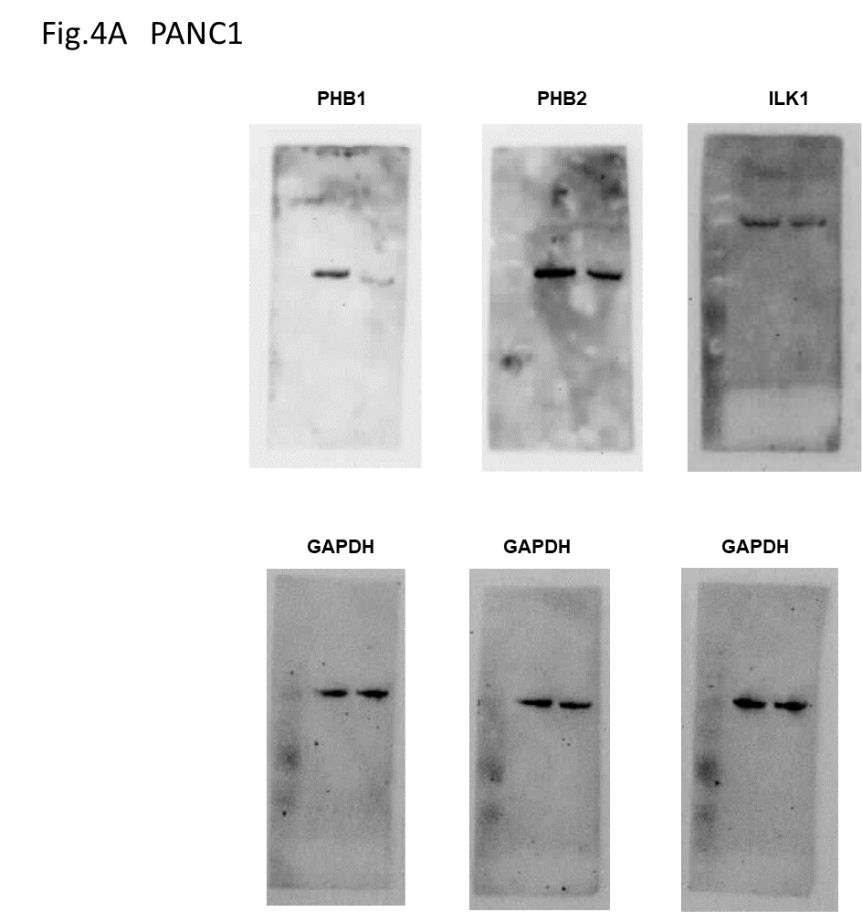
**

**Supplementary Data.**

**Gene ontology analysis of proteins enriched in pseudopodia over cell bodies.**

For over representation analysis, the Fisher exact test was used to investigate an over representation of gene sets (GO Biological processing, GO molecular function, GO cellular components, KEGG pathway). For each protein, the query set was defined as the set of protein groups for which *P < 0.05* for that particular contrast. For each of these query protein groups, the corresponding uniprot identifiers were mapped to entrez gene identifiers using the mappings in the R package [1]. When multiple mappings were available, all possible mappings were used. Finally, this set of query entrez gene identifiers was used for an over representation analysis of GO Biological process, GO molecular function, GO cellular components, and KEGG pathway, all of which contain gene sets defined in terms of their corresponding entrez gene identifiers.

<Reference>

[1] Carlson M (2016). Org.Hs.eg.db: Genome wide annotation for human. R package version 3.4.0.
